# Prevalence of Early Childhood Caries in South Africa: Protocol for a Systematic Review

**DOI:** 10.2196/25795

**Published:** 2021-08-03

**Authors:** Faheema Kimmie-Dhansay, Robert Barrie, Sudeshni Naidoo, Tina Sharon Roberts

**Affiliations:** 1 Division of Research and Postgraduate Studies Faculty of Dentistry University of the Western Cape Cape Town South Africa; 2 Department of Community Dentistry University of the Western Cape Cape Town South Africa; 3 Diagnostics Cluster University of the Western Cape Cape Town South Africa

**Keywords:** dmft, prevalence, dental caries, South Africa, early childhood caries

## Abstract

**Background:**

Young children are at the highest risk of developing dental caries as they have a lack of autonomy over their diet and oral hygiene practices. Dental caries develops over time due to demineralization of tooth substance (enamel), which results from acid production during sugar metabolism by bacteria. Early onset of dental caries often results in asymptomatic presentation, but if left untreated, it can result in severe pain, infection, and dentoalveolar abscesses. Early childhood caries (ECC) is defined as dental caries in children aged 6 years and younger and is a significant public health problem in South Africa. According to the Global Burden of Disease study, untreated dental caries of primary teeth affects 532 million children. Untreated dental caries has many detrimental effects which can affect the physical development and reduce the quality of life of affected children. Furthermore, long-term untreated dental caries can result in school absenteeism, low BMI, and poor educational outcomes.

**Objective:**

The purpose of this study was to determine the prevalence and severity of ECC in South Africa in children under the age of 6 years.

**Methods:**

All cross-sectional studies documenting the prevalence and severity of dental disease (decayed, missing, and filled teeth scores) will be included. Various databases will be searched for eligible studies. Only studies conducted on South African children aged 6 years and under will be included. There will be no restriction on the time or language of publication. The quality of all eligible studies will be analyzed by a risk of bias tool developed by the Joanna Briggs Institute. The results will be presented narratively, and if possible, a meta-analysis will be conducted.

**Results:**

The protocol is registered with PROSPERO. The literature search was initially conducted in November 2018 and was repeated in November 2020.

**Conclusions:**

The results of this study will be used to advise stakeholders of the prevalence and severity of dental disease in children under 6 years of age in South Africa.

**Trial Registration:**

PROSPERO CRD42018112161;

**International Registered Report Identifier (IRRID):**

DERR1-10.2196/25795

## Introduction

Early childhood caries (ECC) is a significant public health problem in children aged 6 years and under living in South Africa [1]. According to the Global Burden of Disease study, the prevalence of untreated dental caries in primary teeth is 532 million [2].

Untreated dental caries has many adverse effects that can affect physical development, including increased absenteeism from school [3], low BMI [4,5], negative educational outcomes [3], and poor oral health-related quality of life [6,7].

Children are at the highest risk of developing dental caries as they are vulnerable and depend on their caregivers for their dietary needs and oral hygiene. Dental caries develops over time and is a consequence of the demineralization of tooth enamel by acids produced during the metabolism of sugars by cariogenic bacterial sugars [8]. The early stages of the disease are often asymptomatic, but if left untreated, dental caries can result in severe pain and life-threatening infections.

Global statistics show an inconsistent prevalence of ECC between different continents and within the same country. In 2007, the prevalence of ECC in children under 5 years of age was 40% in Brazil [9], and in 2016, it varied between 41.9% and 16% in 2 separate districts in India [10,11]. Ismail and Sohn [12] conducted a systematic review in China and reported that the prevalence of ECC in the country was between 78.6% and 85.5%. A later study by Zhang et al [13] recorded prevalence rates between 0.3% and 70.7% in children aged 1-6 years in the same country.

The most recent prevalence rate of ECC in China was documented by Zeng et al [14], who recorded it to be 49.13% in preschool children between ages 3 and 5 years in a southeast Chinese province. Similar varying prevalence rates were recorded across continents (ranging from 22.9% in India to 90% in Indonesia) [15].

African countries have also shown varying prevalence of ECC: In Burkina Faso, Mazza et al [16] recorded a prevalence rate of 16.6% and in Nigeria, Folayan et al [17] reported an ECC prevalence rate of 6.6%. Higher but inconsistent prevalence rates were documented in Sudan (52.4% [18] and 71.4% [19]), whereas in Uganda, 64% of 3-5-year-old children had ECC [20].

In South Africa, the national prevalence rate of ECC is 60% among children under 6 years or age [1]. The prevalence of dental caries in children aged between 2 and 4 years in Johannesburg was 47.74% [21], whereas in the Western Cape, this varied from 71.6% [22] to 80% [23].

For the allocation of resources necessary to manage ECC effectively, it is important to understand the demographics of South Africa. The country is inhabited by 55.7 million people, among which 10.3% are under the age of 5 years [24]. Approximately 20% of children reside with either a grandparent or a caregiver [25], and 13.1% of households live in informal dwellings. Many families lack access to basic amenities including electricity, clean water, food, and a stable income [26] and more than one-quarter of the population rely on social grants, particularly in the poorest provinces [25]. Furthermore, the prevalence of HIV in the country was estimated to be 13.1% in 2018 [24]. With the high level of poverty, lack of access to infrastructure, and high HIV prevalence, the prevention of ECC has not been a priority in this country.

The purpose of this study was to determine the prevalence and severity of ECC in South Africa in children under 6 years of age. To date, this will be the first scientifically conducted systematic review on the prevalence of ECC in South Africa.

## Methods

### Study and Ethics Approval

This protocol will be conducted using the PRISMA-P (Preferred Reporting Items for Systematic reviews and Meta-Analyses for Protocols) guidelines [27]. Ethics approval was not required as this is not a primary study involving participants. The study protocol was registered with PROSPERO (CRD42018112161) on November 21, 2018.

### Study Eligibility Criteria

#### Types of Studies

Cross-sectional and cohort studies reporting the prevalence of ECC in healthy children aged 6 years and under living in South Africa will be included in the review. This is a prevalence/incidence study, and consequently, no interventions will be assessed. The primary outcome is the prevalence/incidence and severity of ECC. The severity of ECC will be measured using the WHO guidelines in infants and children up to the age of 6 years. The WHO criteria include dmft scores (decayed, missing, and filled teeth; lower case indicates deciduous teeth) and the percentage of children that are caries free (including noncavitated caries [white spot lesions]).

#### Information Source and Search Strategy

Databases such as PubMed/MEDLINE, Cochrane, Scopus, Academic Search Complete, Dentistry and Oral Science, CINAHL, and ScienceDirect will be searched. Each database will be examined using tailor-made search terms or MeSH terms: (1) “early childhood caries” OR “caries” OR “decay” OR “dmft” OR “dental” OR “oral” OR PUFA; (2) “prevalence”; (3) “children” OR “peri-natal” OR “paediatric” OR “pediatric” OR neonatal OR infant; (4) South Africa. The keywords were used in the following combinations: 1 + 2 + 3 + 4.

Scientific articles published in all South African official languages will be included in the review. Non-English articles will be translated by the Department of Foreign Languages, University of the Western Cape or a reputable translation company. To authenticate the translations, we will cross-reference the original article with the English abstract (which is usually available online) and reverse translations will be conducted to ensure its correctness.

Commentaries/letters and other gray literature will be excluded from this review.

Secondary searching (PEARLing) will be conducted (PEARLing is a search strategy where the reference lists of all the studies, whether included or excluded, are identified for possible inclusion). Manual searching will not be conducted due to the difficulty in replicating this method.

#### Study Selection

The articles will be uploaded into Rayyan [28] and screened in 2 stages. Two review authors (FK-D and TR) will independently assess the titles and abstracts of the studies and compare them against the inclusion criteria. The full texts of eligible papers and those that contain insufficient information will be sourced.

Other reviewers will be consulted when a disagreement pertaining to the inclusion of a publication arises. The searching process will include all prevalence studies up to November 15, 2020. All eligible studies will be included, and authors will be contacted if any clarification is needed.

After reading the full-text articles, those that do not meet the inclusion criteria will be discarded and the reasons will be recorded in the “Characteristics of excluded studies” table. The reference list of all included publications will be reviewed for additional eligible studies.

### Data Extraction and Management

Two reviewers (FK-D and TR) will independently extract data onto a standardized data extraction form (initially piloted on a small sample of studies) using Microsoft Excel (2014). Upon completion of data collection, the data will be uploaded to the University of the Western Cape’s data repository for safekeeping [29]. The data will be pilot tested, and the independent authors will be trained on how to extract data. The content of the form will be compared, and any differences in opinion will be resolved by discussion and consultation with the other reviewers. If any information from the studies is unclear or missing, the corresponding authors of the original papers will be contacted (where feasible). Study information will include author, title, year of publication, study design, and year in which the study was conducted. Participant-level data will include age, the province where the study was conducted, dmft score and standard deviation, number of cases and total sample size, and urban/rural setting. Pooled prevalence will be obtained by dividing the number of participants with the caries with the number of participants in the whole population, and the data will be assessed using Stata (StataCorp LLC). Pooled standard deviations will be calculated using the Cohen (1998) formula [30].

### Availability of Data and Materials

All data, irrespective of the quality of publication, will be included in the review. If details on study publications cannot be obtained, a librarian will be consulted, and if the study remains non-obtainable, it will not be included in the qualitative or quantitative analysis.

### Study Quality and Risk of Bias Assessment

The quality assessment of studies will be conducted using the Joanna Briggs Institute (JBI) Critical Appraisal Checklist for Studies Reporting Prevalence Data [31].

### Analysis of Study Findings

A meta-analysis will be conducted, using Stata 17, if there are studies of similar comparisons reporting the same outcomes using a random-effects model and only if there are 4 or more studies.

### Assessment of Heterogeneity

This review will include diverse modalities of interventions and will result in heterogeneity of the content of interventions, outcomes, and outcome measures. We will contemplate the feasibility of conducting a meta-analysis on a subgroup of included studies once the data have been extracted. Where feasible, we will assess the statistical heterogeneity in the meta-analysis by visually inspecting the scatter of effect estimates on the forest plots, Cochran test (using .10 level of significance), and by using the *I*^2^ statistic [32].

### Assessment of Reporting Biases

Where possible, we will use multiple sources of data, including those from unpublished trials. Should a meta-analysis be conducted, we will assess publication bias according to the recommendations described in the *Cochrane Handbook for Systematic Reviews of Interventions* [32]. Reporting biases such as selective reporting, duplication, and language of publication will be investigated.

### Analysis of Subgroups or Subsets

We will use a subgroup analysis to examine heterogeneity using Stata 17. This will include exploring the influence of factors such as participant age, province, and urban/rural status. If sufficient numbers of studies are included, a meta-analysis will be performed.

## Results

This protocol was registered with PROSPERO in October 2018, and the electronic searches were completed by November 15, 2020. The original search yielded 2247 articles.

## Discussion

### Principal Results

The study aims to assess the prevalence of dental diseases and its severity in children under the age of 6. The South African government does not regularly monitor the dental disease of children or adults. The last national oral health survey was conducted in 2004 in children only and adults were excluded [33]. There are plans to determine the disease prevalence and severity in South Africa in the next few years. Until then, this review will inform the dental and medical fraternity about the prevalence of ECC in South Africa.

### Conclusions

There are very few studies detailing the prevalence and severity of dental disease in young children in South Africa. It is imperative that we monitor the trends of dental disease in children to inform stakeholders of this burden. Dental disease is a noncommunicable disease, and is associated with childhood obesity and childhood diabetes. More efforts need to be made to prevent the onset of dental disease, and thus prevent the incidence of other noncommunicable diseases in the future leaders of South Africa.

## Abbreviations

ECC: early childhood caries
WHO: World Health Organization
